# Assessing the effectiveness of artificial intelligence education and training for healthcare workers: a systematic review

**DOI:** 10.1186/s12909-026-08969-3

**Published:** 2026-03-10

**Authors:** Leanna Woods, Kayley Lyons, Anton Van Der Vegt, Quita Olsen, Wenyao Huang, Johnson S. Khor, Nancy Xu, Clair Sullivan

**Affiliations:** 1 Queensland Digital Health Centre, Faculty of Health, Medicine and Behavioural Sciences, The University of Queensland, Level 5 Health Sciences Building, Royal Brisbane and Women’s Hospital Campus, Herston, Brisbane, Queensland 4006 Australia; 2 Centre for Digital Transformation of Health, The University of Melbourne, Melbourne, Australia; 3 School of Nursing, Midwifery and Social Work, The University of Queensland, Brisbane, Australia; 4 Faculty of Health, Medicine and Behavioural Sciences, The University of Queensland, Brisbane, Australia; 5 Metro North Hospital and Health Service, Herston, Brisbane, Australia

**Keywords:** Health personnel, Health workforce, Artificial intelligence, Education, Training programs

## Abstract

**Background:**

Artificial intelligence (AI) is increasingly integrated into healthcare, yet upskilling the health workforce remains a challenge. We addressed the research question: *What evidence exists on the effectiveness of AI education and training programs in improving AI literacy among healthcare workers?*

**Methods:**

Following PRISMA guidelines and PROSPERO registration, five databases (PubMed, Scopus, CINAHL, Embase, ERIC) were searched on 20 August 2024, focusing on studies with an intervention of AI training or education for the healthcare workforce, in any study design that reported an evaluation.

**Results:**

27 studies were included. Programs improved AI literacy outcomes mapped to levels 1–3 of the Kirkpatrick-Barr training evaluation hierarchy including improved learner reactions, shifts in attitudes and perceptions, enhanced knowledge and skills, and behavior changes. Programs did not map to level 4, where healthcare workers learn to metacognition levels, including organizational change and patient benefit. Programs were short in length (44%), delivered in academic settings (56%), to doctors (44%) or medical students (44%), at entry-to-practice level (56%). Most taught an introduction to AI (67%), with technical AI skills less frequent.

**Conclusions:**

These programs are a promising start but often lack sufficient depth to build advanced competencies. Improving AI literacy in healthcare will require appropriate course design, an evolving understanding of this rapidly changing area, and evaluating learning effectiveness. As the adoption of AI accelerates across healthcare, health systems may seek to standardise and assess the efficacy of these courses.

**Supplementary Information:**

The online version contains supplementary material available at 10.1186/s12909-026-08969-3.

## Background

Artificial intelligence (AI) in healthcare is increasingly prevalent. AI presents substantial opportunities, such as the possibility of reduced administrative burden and improved care [1]. However, the realization of these benefits is reliant upon active participation of healthcare workers [2]. The level of AI literacy among the current and future healthcare workforce can hinder the integration of AI into practice, and it is unclear how current healthcare training and education programs are supporting an AI literate workforce [3–5]. 

AI literacy refers to the human competencies required to critically adopt, evaluate, interact and use AI tools in various environments [6]. The domains of AI literacy vary in the literature, with three models of AI literacy most relevant. Ng et al. [7] describes four domains of AI literacy: knowing and understanding AI, using and applying AI, evaluating and creating AI, and AI ethics. Almatrafii et al. [8] describes six key constructs: recognize, know and understand, use and apply, evaluate, create, and navigate ethically. Whereas Kong et al. [9] group AI literacy into four dimensions: knowledge and concepts, conceptual understanding, strengths and limitations, and problem solving. There is no single universally agreed framework for AI literacy [6–10]. 

The task of preparing a workforce capable of utilizing healthcare AI to enhance health outcomes remains unresolved [1]. Healthcare students lack exposure to AI education in medicine, radiology, nursing, midwifery and allied health degrees [3, 11–16]. Self-reported AI knowledge is low in medical students [11]. Although students believe that AI is an assistive technology that will lead to improved outcomes in healthcare [17], some are apprehensive about potential negative impacts, expressing concerns of risks such as unemployment, the devaluation of the medical profession, erosion of trust, and negative effects on patient-physician relationships [17]. Generally, attitudes toward AI in healthcare are based on varied and limited knowledge, highlighting a need for standardized and integrated AI education programs [18, 19]. 

Learning programs for healthcare professionals are designed to bridge the gap between technological advancements and practical application [20]. Programs aim to equip current and future healthcare professionals with competencies and literacy in AI, enabling them to harness its capabilities for improved patient outcomes and operational efficiency [21]. Upskilling in new technologies is a global challenge, compounded by a stressed, overworked workforce, insufficient governance frameworks, understandable concerns about data privacy, complex medicolegal implications, and unchecked regulation of technologies [1, 22, 23]. Impactful implementation of education and training initiatives aimed at upskilling the workforce can help mitigate adoption concerns, patient safety risks and foster confidence in AI adoption at scale [24]. 

There is an absence of rigorous and structured evaluation approaches appraising the effectiveness of AI education and training programs [21], particularly regarding secondary outcomes such as organizational change, care delivery and patient benefits [2]. Existing studies also appear to insufficiently address ethical considerations related to AI implementation in healthcare [25]. Current evidence suggests AI training and education programs are largely targeted to single clinical profession groups with less attention to interprofessional practice, which consequently results in unequal AI literacy across the healthcare workforce [5]. Published evidence in this field is limited, with two scoping reviews identified [2, 21], and no systematic reviews on the topic.

### Objectives

Our research question was: *What evidence exists on the effectiveness of AI education and training programs in improving AI literacy among healthcare workers?* The objective was to understand how AI training and education programs designed to improve AI literacy in healthcare workers are implemented and evaluated.

## Methods

### Protocol and registration

The systematic review followed the Preferred Reporting Items for Systematic Reviews and Meta-Analyses (PRISMA) reporting guidelines [26] and adhered to the STORIES statement (STructured apprOach to the Reporting In healthcare education of Evidence Synthesis) [27]. The review protocol followed the PRISMA-P checklist [28] and was registered in PROSPERO (CRD42024575061).

### Eligibility criteria

The target population was the healthcare workforce, which refers to ‘all individuals who deliver or assist in the delivery of health services or support the operation of health care facilities’ [29]. All clinical (e.g., medical doctors, nurses, allied health professionals, pharmacists, Indigenous healthcare workers, pre-registration/qualification students undertaking placements in health care facilities) and non-clinical healthcare workers (e.g., administration, executive and management, clinical support, social work, and volunteers) were considered regardless of professional body or government registration status. Patients or consumers of healthcare were excluded.

The intervention was AI related education or training. AI refers to the application of advanced algorithms, machine learning techniques and machine generated data analytics [30] and excludes traditional digital technologies, general computing and query-based data analysis. Studies were included if they reported the focus of AI was to support healthcare delivery including diagnosis, prevention, prognosis monitoring or treatment of individuals or populations [31]. Training and education relate to initiatives that build AI capability, including programs, curriculum, and courses [32]. Education refers to theoretical learning (e.g., by an academic institution, qualification) and training involves teaching practical skills (e.g., employer-provided professional development, and ‘just-in-time’ training) [29, 33]. AI-related education and training encompasses all formal and informal methods of AI knowledge transmission or learning. It excluded studies primarily focused on enhancing the clinical knowledge of workers even if delivered through AI education tools.

All study designs that reported an evaluation were considered, including randomized controlled trials, non-randomized controlled trials, before-after studies, cohort studies, case-control studies, cross-sectional studies, descriptive studies and reviews. Case reports, development studies, unpublished studies, trial protocols, theses and articles not available in full text or English were excluded. Studies set in healthcare facilities or education and training facilities such as hospitals, primary care settings, universities or vocational training institutions were included. Articles published in English within the last 10 years were considered.

### Search strategy

To identify relevant keywords, an initial search of PubMed was conducted, followed by an analysis of the text words and index terms contained in the title and abstract. This analysis informed the development of a comprehensive search strategy in PubMed with assistance from a medical research librarian (Supplementary Material 1). Four search criteria and their synonyms were combined in this review: (1) AI (2), healthcare workforce (3), education (4), outcomes. The search was replicated in the medical databases Scopus, Cumulative Index for Nursing and Allied Health Literature (CINAHL), and Embase. The education specific database Education Resources Information Centre (ERIC) was also searched. The databases were searched on 20 August 2024. Modifications to searches were not made since registering the protocol.

### Study selection

Search results were uploaded into Covidence review software (Veritas Health Innovation, Melbourne, Australia; www.covidence.org) to remove duplicates and conduct screening. Screening, eligibility and inclusion of studies was undertaken by two independent reviewers against the inclusion criteria. Articles that did not satisfy the criteria were excluded with reasons for exclusion recorded. Any disagreements that arose between the reviewers at each stage of the selection process were resolved through discussion and consensus.

### Data extraction and synthesis

Data extraction was standardized using the data extraction Excel file developed and piloted by the team (Supplementary Material 2). Data was extracted by one reviewer and validated by a second reviewer. From the AI training and education programs, we extracted participant occupation and level of education, setting, trainer details, type of training, length of program and frequency, training topics and assessment methods.

Due to heterogeneity in the study’s author evaluation methods and definitions of AI literacy, a meta-analysis was not feasible; we undertook a narrative synthesis to address the research question. To underpin our synthesis and to establish levels of education effectiveness, we drew upon the Kirkpatrick model [34], modified by Barr et al. 2000 [35] for evaluating training and education programs. The first three levels reflect AI literacy as an individual outcome: (1) learners’ reaction, (2a) modification of attitudes, (2b) acquisition of knowledge and (3) change in behaviour. Higher levels align with AI literacy as an outcome beyond the individual; (4a) change in organizational practice measures and (4b) benefits to clients.

A rapid search of the existing literature in an academic database revealed the absence of a single comprehensive AI literacy framework applicable across all levels of the Kirkpatrick-Barr evaluation model. To address this gap, we developed an integrated AI literacy framework specifically designed for health education and workforce development (Table 1). This framework synthesises key elements from three established models reported by Ng et al. [7], Almatrafi et al. [8], and Kong et al. [9] The selection of these models was informed by a broad but rapid literature search aimed at identifying relevant frameworks. A structured workshop involving two researchers was conducted to critically appraise and select the most suitable models that suited the population and setting of our review. The chosen frameworks were deemed complementary in scope, capturing both established and emerging approaches and collectively provided a comprehensive foundation for constructing the review findings across the continuum of AI literacy in health education.

**Table 1:** Kirkpatrick-Barr model [34] levels for evaluating training and education programs

Level of Kirkpatrick-Barr	Description	AI literacy models
Level 1: learners’ reaction	Participant views of the learning experience and satisfaction with the program	Not applicable
Level 2a: modification of attitudes/perceptions	Changes in reciprocal attitudes or perceptions between participant groups, toward patients/clients and their condition, circumstances, care, and treatment	AI empowerment (AI self-efficacy, meaningfulness, impact and creative self-efficacy) [9] Recognise (be aware) of AI [8]
Level 2b: acquisition of knowledge/skills	Changes in knowledge and skills	Knowing and understanding AI [7,8] AI ethics [7, 8] Using and applying AI (in simulation [7, 8] Evaluating and creating AI (in simulation) [7, 8]
Level 3: change in behaviour	Changes in behaviour transferred from the learning environment to the workplace	Using and applying AI (in practice) [7, 8] Evaluating and creating AI (in practice) [7, 8]
Level 4a: change in organisational practice	Changes in the organization or delivery of care attributable to an education program	Not applicable
Level 4b: benefits to patients/clients	Improvements in the health and well-being of patients/clients as a direct result of an education program	Not applicable

Our synthesis followed a structured three-step process. In Step 1, during data extraction, we mapped the education evaluation methods reported in the included studies to the corresponding levels in the Kirkpatrick-Barr model. For example, “Pre-and post-intervention confidence scores in AI or machine learning (ML) concepts and technical skills” [37] was aligned with level 2 A: Modification of attitudes/perceptions. In Step 2, we conducted an inductive thematic analysis of the mapped entries from Step 1 working within each Kirkpatrick-Barr model level, for example creation of the theme “Greater self-efficacy in AI-related competencies”. This analysis was initially conducted by one researcher, and subsequently checked by a second researcher to ensure consistency and rigour. In Step 3, we examined the alignment between the themes identified in Step 2 and the domains of the AI literacy framework. For example, aligning the theme of “Greater self-efficacy in AI-related competencies” to the AI literacy domain of “AI empowerment”. This step ensured that the educational outcomes extracted from the literature were relevant to AI literacy, in accordance with the focus of the research question. The outcome of this three-step synthesis was a consolidated list of AI literacy education and training outcomes, each mapped to both the Kirkpatrick-Barr evaluation levels and the corresponding domains of the AI literacy framework.

To support implementation of AI training and educational programs, we extracted barriers and enablers for implementing AI training and education for healthcare workers. Comments from learners and author statements were captured. The synthesis involved an inductive analysis of relevant text segments from individual studies, with themes generated across several workshops among two researchers until consensus was achieved.

### Quality assessment

The quality of included studies was assessed using the mixed-methods appraisal tool (MMAT) [36], for systematic reviews that includes empirical qualitative, quantitative (randomized controlled trial, non-randomized and quantitative descriptive) and mixed method study designs. Five criteria were assessed for each study design by two independent reviewers and disputes resolved by consensus. No study was excluded from the review based on the MMAT score.

## Results

### Characteristics of included studies

**Fig. 1:**
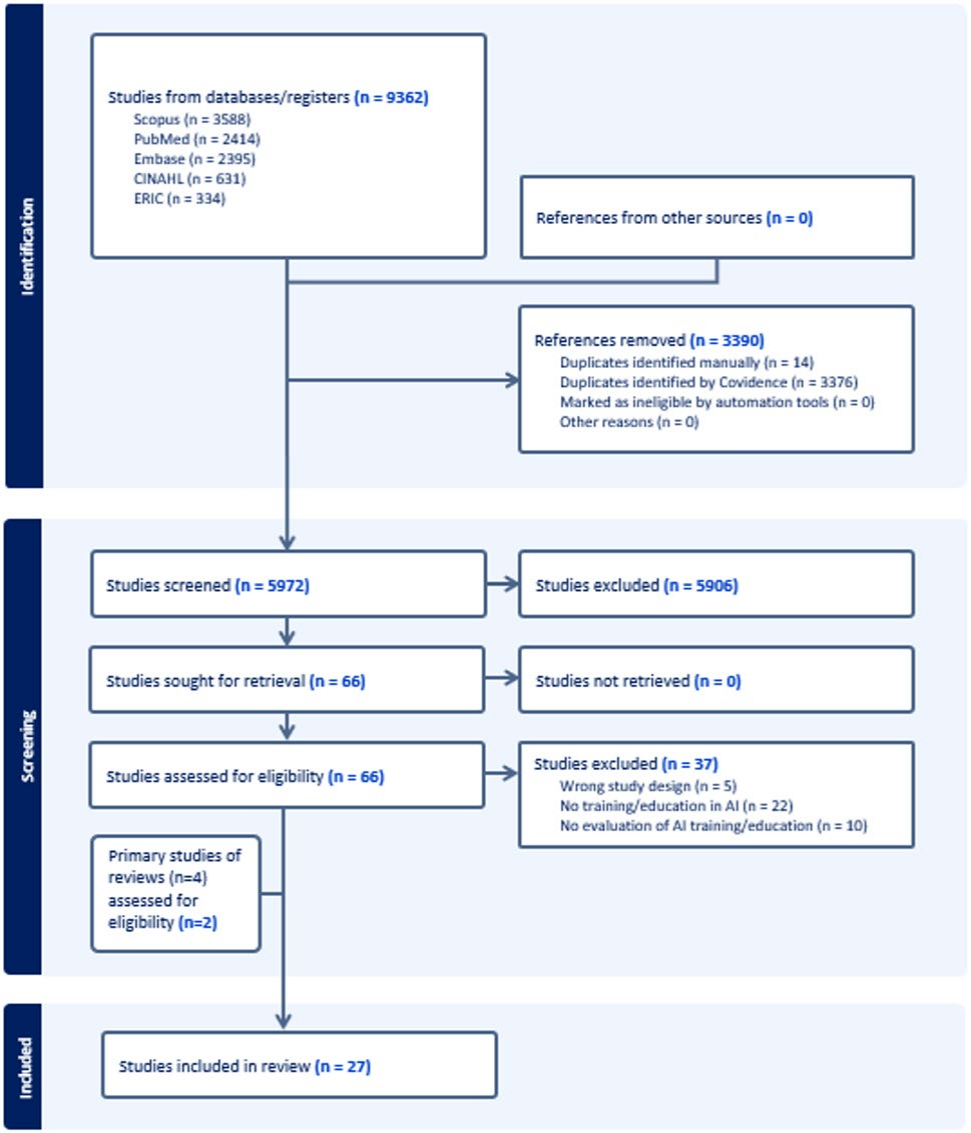
PRISMA flow diagram

Of the 9632 studies identified, 66 were assessed for full text eligibility and 37 were excluded, resulting in 27 included studies (Fig. 1) [37–63]. The main reason for exclusion was the absence of an education or training intervention (*n* = 22). Included studies were conducted in 10 countries and comprised approximately 2160 learners (ranging from 3 [63] to 395 [53] learners) (Table 2). Most studies were conducted in the USA (*n* = 9) [37, 38, 40–43, 50, 52, 63] and its territory Puerto Rico (*n* = 4) [47, 55–57]. All studies were cross sectional with mixed methods (*n* = 10), quantitative non-randomized (*n* = 10), qualitative (*n* = 5), quantitative descriptive (*n* = 1) and non-randomized before after (*n* = 1) study designs.

**Table 2:** Article summary table

Author, year, country	Education intervention	Study type; design	Evaluation approach	Sample size
Abid, 2024, USA [37]	Self-guided, web-based course	Mixed methods	Framework: Not reported Evaluation: Pre- and post-elective survey	19
Barbour, 2019, USA [38]	Half-Day Educational Summit	Quantitative non-randomized	Framework: Not reported Evaluation: Pre- and post-survey	Not reported
BinJalal, 2024, UK/International [39]	Virtual Course: 2-day digital health literacy course	Mixed methods	Framework: Acceptability of Intervention Measure Evaluation: Pre- and post-survey	62 (33 participants, 29 waitlist controls)
Bumback, 2024. USA [40]	Assignment with ChatGPT written guidelines provided	Qualitative	Framework: Not reported Evaluation: ChatGPT assessment	113 total (qual = 2)
Chadha, 2024, USA [41]	A 60 to 90-minute faculty development workshop on generative AI	Mixed methods	Framework: Not reported Evaluation: Pre- and post-workshop survey	36
Culp, 2024, USA [42]	Didactic lecture as well as a 2-day practice project done in small groups	Quantitative non-randomized	Framework: Not reported Evaluation: Pre- and post-survey	> 170
Finkelstein, 2024, USA [43]	Certificate Course − 6 online modules with end of module quizzes and pre/post assessments	Quantitative non-randomized	Framework: Not reported Evaluation: Pre- and post-course assessments, post-course survey	42
Franco D’Souza, 2024, India [44]	Multi-day workshop	Mixed methods	Framework: Not reported Evaluation: Pre- and post-workshop questionnaires and focus group discussions	Not reported
Griewing, 2024, Germany [45]	30 min educational presentation about the application of artificial intelligence and blockchain in managing breast cancer	Quantitative non-randomized	Framework: Not reported Evaluation: Pre- and post-questionnaire	45 pre-education survey, 30 post-education survey
Hedderich, 2021, Germany [46]	12-week online-only course	Mixed methods	Framework: Not reported Evaluation: Pre- and post-course questionnaires and group work task	93 total (28 completed course)
Heredia-Negron, 2024, USA (Puerto Rico) [47]	Online 3-month course	Quantitative non-randomized	Framework: Not reported Evaluation: Pre- and post-test after course completion	50
Hu, 2023, Canada [48]	3-week virtual course without attendance recorded	Quantitative non-randomized	Framework: Bloom’s Taxonomy of Learning Evaluation: Pre- and post-workshop survey	12 total (11 provided survey responses)
Kansal, 2022, India [49]	Two free webinars	Quantitative descriptive	Framework: Not reported Evaluation: Post-webinar questionnaire	621 total (367 completed questionnaire)
Krive, 2023, USA [50]	4-week modular AI course delivered online with small group active learning	Mixed methods	Framework: Not reported Evaluation: Pre- and post-quiz and reflections	20
Laupichler, 2022, Germany [51]	A flipped classroom course with online self-study units and online classroom lessons	Quantitative non-randomized	Framework: Medical Artificial Intelligence Readiness Scale for Medical Students (MAIRS-MS) Evaluation: Pre- and post-quizzes and general assessments	24
Lindqwister, 2021, USA [52]	An integrated artificial intelligence curriculum (AI-RADS): lectures and journal club	Quantitative non-randomized	Framework: Not reported Evaluation: Pre- and post- lecture survey	12 initial (5 at last lecture)
Mishra, 2023, India [53]	3-month course with lectures and discussions	Quantitative non-randomized	Framework: Not reported Evaluation: Pre- and post-questionnaire	395
Pauwels, 2021, Brazil [54]	1-hour introductory lecture	Quantitative non-randomized	Framework: Not reported Evaluation: Pre- and post-survey	293 questionnaires collected
Perchik, 2023, USA (Puerto Rico) [55]	A week-long AI in radiology course. Two 30- minute lectures were held each day on Zoom	Quantitative non-randomized	Framework: Not reported Evaluation: Pre- and post-survey	Avg. 75 participants each course day (range: 50–120)
Reading Turchioe, 2024, USA (Puerto Rico) [56]	Face to face (didactic lectures) and laboratory sessions	Mixed methods	Framework: Bloom’s Taxonomy of Learning Evaluation: Post-assessment reflections	10 (1 didn’t complete survey)
Richardson, 2022, USA (Puerto Rico) [57]	Three 2-hour face to face classes with pre-class homework. Short didactic learning component (10–15 min) then hands on DL modelling	Mixed methods	Framework: Not reported Evaluation: Post-course survey	20 (11 participated in evaluation)
Taskiran, 2023, Turkey [58]	Three 2-hour face to face classes with pre-class homework. Short didactic learning component (10–15 min) then hands on DL modelling	Quantitative non-randomized	Framework: Medical Artificial Intelligence Readiness Scale (MAIRS) Evaluation: Post-course survey	300
Teferi, 2024, Canada [59]	The program incorporates both synchronous and asynchronous elements to facilitate interactive learning experiences.	Qualitative	Framework: Health Equity and Inclusion theoretical framework; and the Analysis, Design, Development, Implementation and Evaluation (ADDIE) instructional design model Evaluation: Case studies, capstone projects and reflective learning	12
Tspora, 2023, France [60]	Standalone formal training with support to develop and design an AI-CDSS to solve a medical issue, in person	Mixed methods	Framework: Kern’s approach for curriculum development Evaluation: Small group design projects and report	15
Van de Venter, 2023, UK [61]	Virtual Postgraduate-level (masters level; level 7) introductory module on AI for radiographers.	Qualitative	Framework: Social constructivist epistemology and relativist ontology underpinned analysis Evaluation: Written essay and oral presentation	13
Van Kooten, 2024, The Netherlands [62]	Mandatory, formal training for radiologists: 3-day AI curriculum	Mixed methods	Framework: Not reported Evaluation: Pre- and post-curriculum surveys	12
Wiggins, 2020, USA [63]	Self-determined study elective in 4th-year of radiology residency	Qualitative	Framework: Not reported Evaluation: Milestone and competency review, and self-assessment	3

### Quality of included studies

The MMAT critical appraisal tool assessed that eight of the 27 studies [42, 43, 47, 49, 50, 53, 61, 62] were of high methodological quality, with clear research aims and adherence to most criteria for their respective study designs (Supplementary Material 3). Quantitative non-randomized studies generally scored higher than mixed method or qualitative studies. Three studies had unclear data collection related to their research questions [38, 56, 57]. 

### Effectiveness of AI education programs in enhancing AI literacy

We distinguished 15 distinct AI literacy education program effectiveness outcome categories from 25 of the 27 included studies (Table 3). All effectiveness outcome categories were able to be mapped to AI literacy frameworks and to levels within the Kirkpatrick-Barr model. Two studies met the inclusion criteria, but evaluation was limited to acknowledgement that assignments were submitted [40] or had a minimal post course survey [49] so was excluded from Table 3.

**Table 3:** Reported effectiveness of AI education interventions on AI literacy and mapped to the Kirkpatrick-Barr levels for evaluating training and education programs [35] as well as mapped to existing AI literacy model domains [7–9]

Kirkpatrick Barr Level	Effectiveness categories used by studies*	References													
Level 1: learners' reaction (n=19)	Reported satisfaction with the AI education program	[37]	[39]	[41]	[42]	[43]	[46]	[51]	[52]	[57]	[59]	[60]	[61]	[62]	[63]
	Participants perceived that they learned	[38]	[42]	[46]	[47]	[48]	[52]	[53]	[56]	[59]	[60]	[61]	[62]	[63]	
	Significant participant dropout rate	[46]													
Level 2a: modification of attitudes or perceptions (n=21)	Greater beliefs on meaningfulness and impact of AI	[38]	[42]	[44]	[47]	[50]	[53]	[54]	[58]						
	Greater self-efficacy in AI-related competencies	[37]	[39]	[46]	[48]	[51]	[52]	[58]	[62]						
	Increased willingness to use AI	[38]	[41]	[53]											
	Greater awareness and familiarity with AI	[43]	[53]	[55]											
	Increased interest or willingness to learn more about AI	[43]	[55]	[60]											
	Decreased concern about AI	[54]													
	Mixed results about willingness to learn more about AI	[57]													
	No change in willingness to use AI	[45]													
Level 2b: acquisition of knowledge or skills (n=6)	Improved understanding of AI as measured by a test	[39]	[43]	[50]											
	Greater application of AI skills as measured by a performance test	[50]	[56]	[57]											
	Improved understanding of AI ethics as measured by a test	[44]													
Level 3: change in behavior (n=2)	Applied new AI knowledge and skills in an authentic practice	[60]	[63]												
Level 4a: change in organizational practice (n=0)	None	None													
Level 4b: benefits to patients/clients (n=0)	None	None													

*Inductively categorized. When statistics were reported, only statistically significant results were included

^Authors mostly used unvalidated self-efficacy scales; rather they measured confidence or readiness

AI literacy outcomes included improved learner reactions, shifts in attitudes and perceptions, enhanced knowledge and skills, and behavioral changes that translated into authentic workplace settings. Pre- and post-intervention surveys frequently demonstrated that the learners had increased awareness of AI, self-efficacy, understanding, willingness to learn, perceived meaningfulness, and perceived impact. Several studies reported that the learners had heightened willingness to adopt AI and a reduction in AI-related concerns.

The studies employed mostly lower-level Kirkpatrick-Barr levels, especially Level 1 and 2a evaluation approaches. In aligning with the Kirkpatrick-Barr model, 19 studies measured education effectiveness at Level 1, 21 studies at Level 2, 2 studies at Level 3 and no studies at Level 4. Considering the two studies that evaluated effectiveness of their AI education programs at Level 3, both [60, 63] reported that their educational interventions led to tangible workplace behaviors, such as learners engaging in AI research, initiating workplace projects, and pursuing advanced training or academic opportunities like internships and PhD programs.

### AI literacy training and education characteristics

**Fig. 2:**
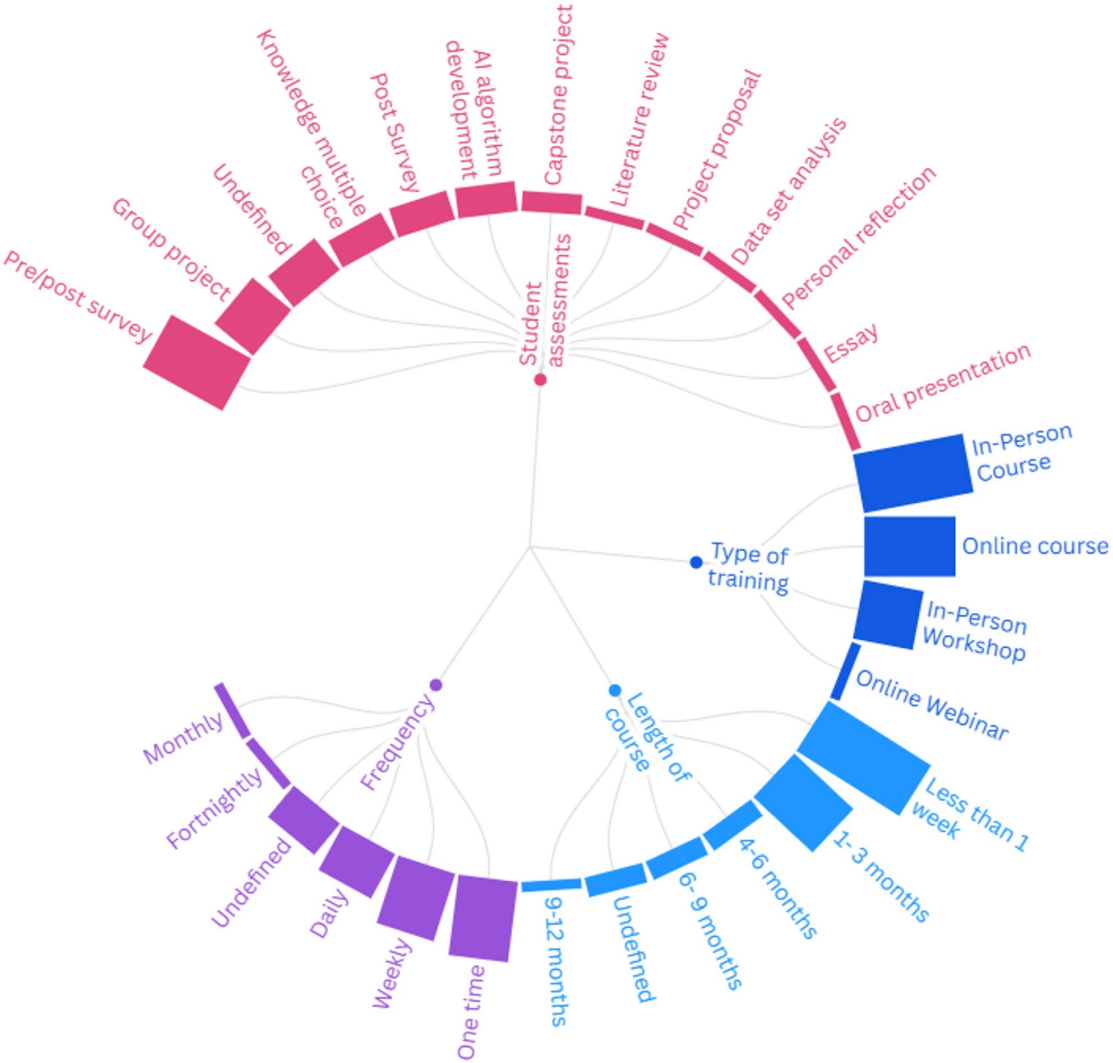
Course delivery characteristics of AI training and education programs in included studies. Bar height indicates relative frequency of studies for that characteristic

AI literacy training course details varied across included studies (Fig. 2 and Supplementary Material 4, interactive online map [64]). Student assessments in the courses showed variability with most assessments being a survey: pre/post course survey (33%) and post course survey (11%). Five studies assessed learners with a group project and three with multiple choice questions (MCQ). Some studies emphasized technical AI development: developing AI algorithms (11%), capstone projects (7%), project proposals (4%) and data set analyses (4%). For instance, in Richardson et al. [57] radiology residents were trained in programing and learned to train deep learning (DL) models. Four studies did not define how their learners were assessed [45, 49, 58, 61]. The majority of AI training or education were in person courses (*n* = 11) or online courses (*n* = 9). Most course duration was less than a week (44%) or between 1 and 3 months (30%) with very few education programs more than 6-month duration (11%). The frequency of education or training varied with most regular sessions conducted weekly (26%) or daily (19%) unless AI training was a one-time session (30%).

**Fig. 3:**
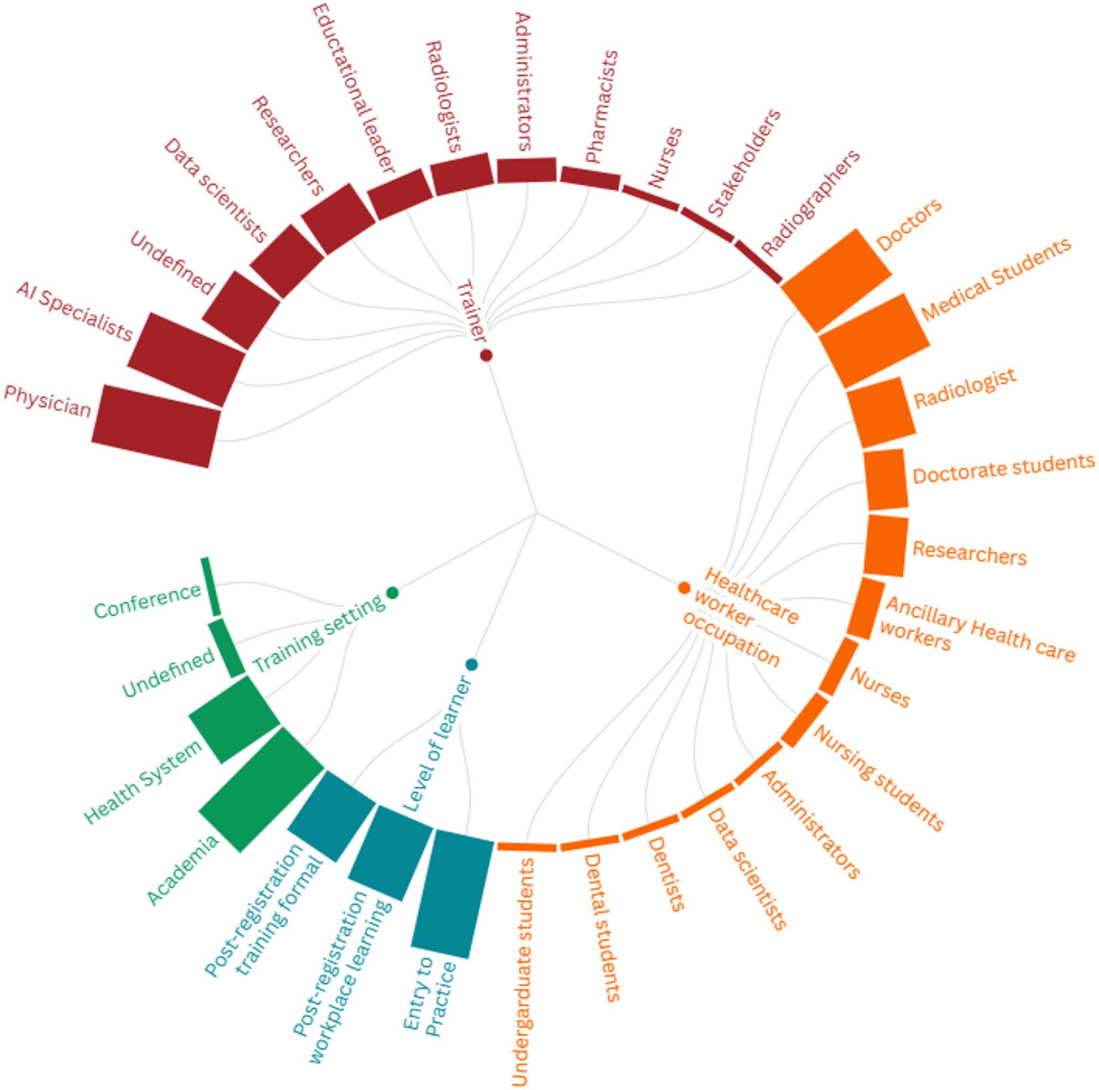
Learner and teacher characteristics of AI training and education programs in included studies. Bar height indicates frequency of studies for that characteristic

Learners and teachers of AI training and education initiatives were largely medical focused, delivered to entry-to-practice healthcare workers (Fig. 3, interactive online map [65]). The occupations of AI learners were mostly doctors (44%), medical students (44%) and radiologists (26%) and one study described learners as undergraduate students, dentists and dental students [54]. The career level of AI learners was largely at entry-to-practice level (56%) with programs also targeted at the post-registration level in the workplace (37%) or in formalized education programs (33%). Education and training was predominantly delivered in academic settings (56%) or within the health system (33%).

### AI topics taught

**Fig. 4:**
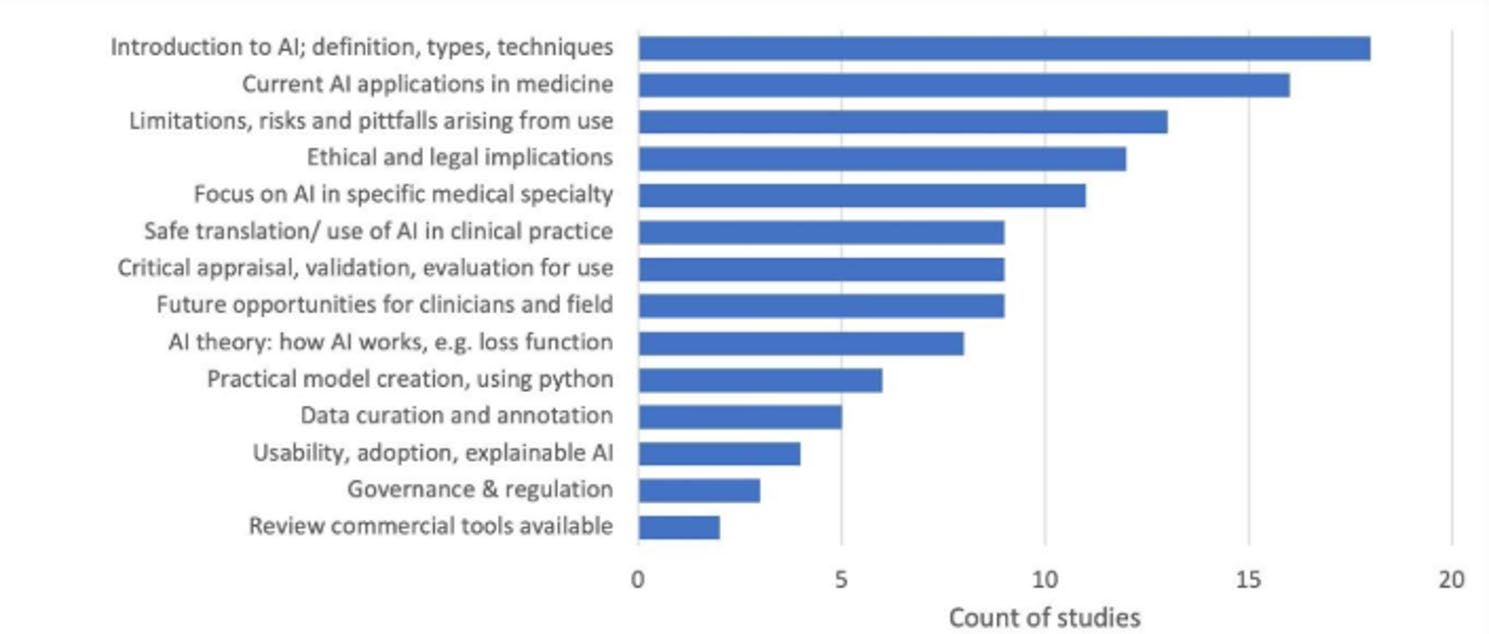
Topics of AI taught to healthcare workers in included studies

There were 14 broad themes taught in the AI training and education topics with most studies (67%) offering an introduction to AI (Fig. 4). The median number of education or training topics per study was five and many studies focused all or a portion of their content on a specific medical specialty: radiology (30%), medical imaging in general (22%), pharmacy (11%) and neuroscience (11%). Some studies addressed the limitations and risks of AI (48%), the ethical and legal implications (44%) and governance and regulation of AI (11%). The safe use of AI in clinical practice, critical appraisal and evaluation AI topics were covered in 33% of courses. Technical AI skills topics were more infrequent: AI theory (30%), model creation with coding (22%), data curation (19%) and reviewing existing AI tools (7%).

### Barriers and enablers to AI training and education programs

In total, 20 distinct enablers and 21 distinct barriers to implementing AI training and education for healthcare workers were identified from the studies (Table 4). Primary enabler categories were appropriate course design, generating interest for future career and benefits to healthcare outcomes. The primary barrier categories were concerns about non-standardization and the complexity of AI education, participant time constraints, lack of processes for implementing AI, negative career impact of AI and the variability AI would have on healthcare outcomes.

**Table 4:** Barriers and enablers to implementing AI training and education programs for healthcare workers. *n* = 27 studies

Enablers	
Appropriate course design (*n* = 15)	Mandatory, formal education or training embedded into curricula [46, 49, 54, 58, 61, 63]
	Good pedagogical design that is detailed and considers complexity [37, 43, 46, 50, 51, 61]
	Sufficient practical learning [37, 41, 57, 63]
	Flexible [37, 51, 61, 63]
	Multidiscipline training and education [50, 59, 61, 62]
	Journal club and post course availability [52, 61, 62]
	Collaborative learning [61, 62]
	Interactive elements [43]
	Technical training and education [59]
	Strong introduction to AI [61]
	Literature appraisal [61]
	Smaller class sizes [62]
	Region specific training and education [62]
Generating interest in future career (*n* = 5)	Motivation to learn [37, 48] Positive career impacts [49, 54] Generates interest in AI [54, 55]
Benefits to healthcare outcomes (*n* = 2)	Inclusive [44] Triaging [44] Improved resources [44] Discipline specific benefits [54]
Barriers	
Non-standardized, complex AI training and education (*n* = 12)	Complexity of AI training and education including rapid changes [48, 56, 58, 61, 62]
	Limited course setting (online) [46, 55, 57, 61, 62]
	Training material not meeting education needs [57, 59, 62]
	Lack of existing training and education [44, 49]
	Learners felt unprepared prior to course [49, 62]
	Lack of interest in technical skills of AI [57]
	Discrepancy between trainers and software knowledge [42]
	Lack of collaborative training and education [62]
Time constraints (*n* = 7)	Scheduling, sequence and time of tasks [37, 46, 50, 52, 61]
	Not enough time to complete requirements [37, 48, 60]
	High dropout rate [46]
Lack of processes for AI implementation (*n* = 4)	Limited existing knowledge of AI [42, 48]
	Unprepared for shift to AI [44, 54]
	Need for regulation, policies and ethics [44]
	Resistance to change [42]
Negative impact on career (*n* = 4)	AI will impact career negatively [38, 54, 58] Not perceived as important [42]
Variable impacts of AI on healthcare (*n* = 3)	Less humanistic [38] Accuracy of AI [41] Privacy [41] AI unable to manage complexity [44]

## Discussion

In an era of rapid technological advancements, AI has emerged as a potentially transformative force in healthcare, necessitating a complementary effort to produce a confident workforce equipped with AI literacy [66]. Our systematic review of 27 studies revealed that current AI education and training programs primarily focus on introductory AI knowledge and attitude development rather than extending further to advanced capabilities. Most programs were short-term and delivered in academic settings to medical professionals. Evaluations were most often mapped to the lower levels of the Kirkpatrick-Barr framework (e.g., learner reactions) and none to level 4 (change in organizational practice or benefits to patients/clients). Although most evidence for AI education and training was generally positive, many programs reported in included studies did not report higher-order competencies including using, applying, creating, and evaluating AI. While the heterogeneity in outcome measures and high proportion of self-reported measures should be considered when interpreting these results, we have revealed a gap and opportunity for more comprehensive approaches to teach healthcare professionals how to effectively use AI in clinical practice.

Our results identified that many (44%) AI education and training programs were less than one week in duration and 30% were delivered as one-time sessions. While these short programs are a positive first step towards workforce development in clinical AI, they do not yet address the comprehensive education and training practices needed to teach healthcare workers more advanced competencies [67]. More rigorous capability development requires adaptability and ongoing learning in complex environments, which are particularly relevant for AI literacy [67, 68]. To support meaningful AI literacy across the healthcare workforce, educators could consider creative, practical and feasible ways to integrate AI as an evolving technology into curriculum design and delivery, tailored to the needs of time-constrained healthcare professionals. Future educators could consider the development of longitudinal, theoretically-grounded educational programs that provide healthcare professionals with opportunities for skill application in authentic clinical contexts [67]. 

The absence of a discoverable comprehensive framework for measuring and characterising AI literacy and fluency in the literature presented a challenge in this review. We began our data analysis with the widely cited Ng et al.’s AI literacy framework [7], however, it had limitations in its conceptual breadth. For example, while Ng et al.’s framework provided a foundation, study findings required additional constructs such as willingness to use AI and interest in learning more about AI, critical dimensions absent from the Ng et al. model. Ng et al.’s framework heavily relies on a reapplication of Bloom’s taxonomy from the 1950s with minimal integration of advances in educational theory such as the inclusion of attitudinal or mindset shifts, which are necessary for understanding readiness and adaptability in AI contexts. Despite extensive searches, no other single framework provided a comprehensive AI literacy construct and as a result, we synthesised findings using three distinct AI literacy models to create a comprehensive framework that covered our study results [7–9]. This account aligns with broader reflections in the field of educational measurement, where frameworks are increasingly required to represent the multidimensional nature of competency development, including attitudes, knowledge, and behaviours [69, 70]. 

Additionally, we encourage AI literacy researchers to consider metacognitive dimensions in their frameworks that encompass self-regulation, reflection, and the ability to adapt one’s learning strategies. This was missing from all reviewed AI literacy frameworks and our study results. The absence of metacognition further limits the utility of current constructs in AI literacy, as highlighted by Flavell’s foundational work on metacognition and its role in effective learning [71]. Our findings highlight the need for a more robust and comprehensive AI literacy frameworks that can holistically measure AI literacy and fluency while incorporating modern insights from educational theory and AI-specific competencies.

There was a notable absence of the use of established AI education evaluation, or competency measurement frameworks in the reviewed studies. First, most authors developed their own constructs, tests, and surveys without utilizing models with evidence of validity, reliability, or generalizability. The lack of standardization of evaluations limits the ability to compare findings across studies. This practice also increases the risk of multiple types of measurement errors, including construct underrepresentation and method bias, which can compromise the validity and reliability of results [72]. Second, the lack of alignment with frameworks that encompass attitudes, skills, knowledge, and behaviours reflects the broader challenge in health professions education, where the application of theories remains inconsistent [73, 74]. This gap in theoretical underpinning impedes the development of robust evidence bases for educational interventions to advance the field. Adopting and adapting well-established educational frameworks can ensure more reliable evaluations of educational outcomes and support the advancement of best practices in AI education for healthcare professionals.

As AI becomes more integrated into healthcare practice, healthcare will need leaders who can look ahead towards new AI enabled models of care and act accordingly [68]. Distinctions will need to be made between which healthcare workers will need to be competent in AI to enable acceptance and adoption and which healthcare worker subgroups will need to be capable in AI to enable effective use to solve healthcare challenges. Such planning will inform curriculum development in the education sector and be necessary for strategic workforce planning in the healthcare sector. Future research could focus on the level of understanding required for different healthcare worker groups and examine the value of deeper levels of understanding in AI on healthcare practice.

### Strengths and limitations

To the best of our knowledge, this is the first systematic review that evaluates the effectiveness of AI education and training interventions for healthcare workers. A strength of this review is the broad search strategy used across five different academic databases. We chose a systematic literature review design and excluded grey literature to focus on published evidence. However, there are some limitations. First, some studies provided limited information on how the intervention was evaluated or were limited by self-reported measures and small sample sizes. Second, there was significant heterogeneity among studies regarding how AI literacy was considered and education outcomes were measured. To account for these differences, we categorised the literacy outcomes inductively and categorised them against three established AI literacy frameworks. Accordingly, some important nuances may have been overlooked in synthesizing the results and limits the ability to draw clear conclusions. Third, the review only includes articles in English.

## Conclusions

With recent advancements in healthcare AI, we are at the beginning of the AI revolution that promises meaningful contributions to healthcare. If health systems and health consumers are to see the benefits of healthcare AI in practice, healthcare workers will need to accept, embrace and lead this movement. The balance between what ‘could be’ and what ‘should be’ in clinical healthcare AI will likely be navigated by healthcare workers who are integrating AI into their practice and driving this change in our health services as change leaders. Evidence on how to effectively educate and train our current and future healthcare workforce in AI is only just beginning to emerge. While these short programs are a positive first step towards workforce development in clinical AI, they appear to not yet meet the comprehensive education and training practices needed to teach healthcare workers more robust competencies. Appropriate course design and an evolving understanding of this rapidly changing area may address implementation barriers. Supporting healthcare worker AI literacy will likely require advances in pedagogical quality of AI training and education programs, suitably measuring learning outcomes and adapting established educational frameworks to evaluate learning effectiveness.

## Supplementary Information

Supplementary Material 1.

Supplementary Material 2.

Supplementary Material 3.

Supplementary Material 4.

## Data Availability

All data generated or analysed during this study are included in this published article [and its supplementary information files].

## Abbreviations

ADDIE: Analysis, Design, Development, Implementation and Evaluation - instructional design model
AI: Artificial Intelligence
CINAHL: Cumulative Index for Nursing and Allied Health Literature
ERIC: Education Resources Information Centre
MAIRS-MS: Medical Artificial Intelligence Readiness Scale for Medical Students
MCQ: Multiple Choice Questions
ML: Machine Learning
MMAT: Mixed-Methods Appraisal Tool
PRISMA: Preferred Reporting Items for Systematic Reviews and Meta-Analyses
PRISMA-P: Preferred Reporting Items for Systematic Reviews and Meta-Analyses -Protocols
STORIES: STructured apprOach to the Reporting In healthcare education of Evidence Synthesis
