# Supplementary material for: Assessing the effectiveness of artificial intelligence education and training for healthcare workers: a systematic review
Source: BMC Med Educ. 2026 Mar 10;26:549. doi: 10.1186/s12909-026-08969-3 (PMC13045066; doi:10.1186/s12909-026-08969-3)
Supplement: Supplementary file 1 — Supplementary Material 1. [file 12909_2026_8969_MOESM1_ESM.docx]

## **Additional file 1 - Search strings**

PubMed Search

| Search number | Search string | Result numbers |
| --- | --- | --- |
| 1 | "Artificial Intelligence"[MeSH] OR "Artificial Intelligence"[Title/Abstract] OR “AI”[Title/Abstract] OR “Deep Learning” [Title/Abstract] OR “Machine Learning” [Title/Abstract] | 360,663 |
| 2 | "education"[MeSH] OR "educat*"[Title/Abstract] OR "curricul*"[Title/Abstract] OR "professional development"[Title/Abstract] OR "teach*"[Title/Abstract] OR “course” [Title/Abstract] OR “fellowship” [Title/Abstract] OR “seminar” [Title/Abstract] OR “workshop series”[Title/Abstract] | 2,214,360 |
| 3 | "Health Personnel"[MeSH Terms] OR "health-care work*"[Title/Abstract] OR "health* work*"[Title/Abstract] OR "health-care staff"[Title/Abstract] OR "health* staff"[Title/Abstract] OR "health-care personnel"[Title/Abstract] OR "health* personnel"[Title/Abstract] OR "health-care professional*"[Title/Abstract] OR "health* professional*"[Title/Abstract] OR "clinician*"[Title/Abstract] OR "health-care student*"[Title/Abstract] OR "health* student*" [Title/Abstract] OR "practitioner*"[Title/Abstract] OR "radiolog*" [TiAB] OR "physiotherapist*"[Title/Abstract] OR "physical therapist*"[Title/Abstract] OR "pharmac*"[Title/Abstract] OR "technician*"[Title/Abstract] OR "physician*"[Title/Abstract] OR "clinical staff"[TiAB] OR "doctor*"[Title/Abstract] OR "medical staff"[Title/Abstract] OR "nurse*"[Title/Abstract] OR "radiotherapist*"[Title/Abstract] OR "midwi*"[Title/Abstract] OR "radiographer*"[Title/Abstract] OR “fellow”[Title/Abstract] OR “resident”[Title/Abstract] OR “intern”[Title/Abstract] | 3,226,789 |
| 4 | “survey*”[Title/Abstract] OR “evaluat*”[Title/Abstract] OR “interview*”[Title/Abstract] OR “questionnaire*”[Title/Abstract] OR “assess*”[Title/Abstract] OR “impact”[Title/Abstract] OR “validat*”[Title/Abstract] | 9,740,040 |
| 1 AND 2 AND 3 AND 4 |  | 2835 |
|  | Filters: in the last 10 years, English | 2,413 |

### Embase Search

| Search number | Search string | Result numbers |
| --- | --- | --- |
| 1 | 'Artificial Intelligence'/exp OR 'Artificial Intelligence':ti,ab OR AI:ti,ab OR 'Deep Learning':ti,ab OR 'Machine Learning':ti,ab | 328,958 |
| 2 | education/exp OR educat*:ti,ab OR curricul*:ti,ab OR 'professional development':ti,ab OR teach*:ti,ab OR course:ti,ab OR fellowship:ti,ab OR seminar:ti,ab OR 'workshop series':ti,ab | 3,242,838 |
| 3 | 'Health Personnel'/exp OR 'health-care work*':ti,ab OR 'health* work*':ti,ab OR 'health-care staff':ti,ab OR 'health* staff':ti,ab OR 'health-care personnel':ti,ab OR 'health* personnel':ti,ab OR 'health-care professional*':ti,ab OR 'health* professional*':ti,ab OR clinician*:ti,ab OR 'health-care student*':ti,ab OR 'health* student*':ti,ab OR practitioner*:ti,ab OR radiolog*:ti,ab OR physiotherapist*:ti,ab OR 'physical therapist*':ti,ab OR pharmac*:ti,ab OR technician*:ti,ab OR physician*:ti,ab OR 'clinical staff':ti,ab OR doctor*:ti,ab OR 'medical staff':ti,ab OR nurse*:ti,ab OR radiotherapist*:ti,ab OR midwi*:ti,ab OR radiographer*:ti,ab OR fellow:ti,ab OR resident:ti,ab OR intern:ti,ab | 5,032,641 |
| 4 | survey*:ti,ab OR evaluat*:ti,ab OR interview*:ti,ab OR questionnaire*:ti,ab OR assess*:ti,ab OR impact:ti,ab OR validat*:ti,ab | 13,290,168 |
| 1 AND 2 AND 3 AND 4 |  | 4,433 |
|  | Filters: in the last 10 years, English, ('article'/it OR 'article in press'/it OR 'conference paper'/it OR 'conference review'/it OR 'review'/it) Not conference abstracts, preprints, letter, note, editorial, chapter, tombstone, erratum, short survey | 2,395 |

### CINAHL search

| Search number | Search string | Result numbers |
| --- | --- | --- |
| 1 | (MH "Artificial Intelligence+") OR (TI "Artificial Intelligence" OR AB "Artificial Intelligence") OR (TI AI OR AB AI) OR (TI "Deep Learning" OR AB "Deep Learning") OR (TI "Machine Learning" OR AB "Machine Learning") | 59,334 |
| 2 | (MH education+) OR (TI educat* OR AB educat*) OR (TI curricul* OR AB curricul*) OR (TI "professional development" OR AB "professional development") OR (TI teach* OR AB teach*) OR (TI course OR AB course) OR (TI fellowship OR AB fellowship) OR (TI seminar OR AB seminar) OR (TI "workshop series" OR AB "workshop series") | 1,417,569 |
| 3 | (MH "Health Personnel+") OR (TI "health-care work*" OR AB "health-care work*") OR (TI "health* work*" OR AB "health* work*") OR (MH "health-care Health Personnel"+") OR (TI "health-care work*" OR AB "health-care work*") OR (TI "health* work*" OR AB "health* work*") OR (TI "health-care staff" OR AB "health-care staff") OR (TI "health* staff" OR AB "health* staff") OR (TI "health-care personnel" OR AB "health-care personnel") OR (TI "health* personnel" OR AB "health* personnel") OR (TI "health-care professional*" OR AB "health-care professional*") OR (TI "health* professional*" OR AB "health* professional*") OR (TI clinician* OR AB clinician*) OR (TI "health-care student*" OR AB "health-care student*") OR (TI "health* student*" OR AB "health* student*") OR (TI practitioner* OR AB practitioner*) OR (TI radiolog* OR AB radiolog*) OR (TI physiotherapist* OR AB physiotherapist*) OR (TI "physical therapist*" OR AB "physical therapist*") OR (TI pharmac* OR AB pharmac*) OR (TI technician* OR AB technician*) OR (TI physician* OR AB physician*) OR (TI "clinical staff" OR AB "clinical staff") OR (TI doctor* OR AB doctor*) OR (TI "medical staff" OR AB "medical staff") OR (TI nurse* OR AB nurse*) OR (TI radiotherapist* OR AB radiotherapist*) OR (TI midwi* OR AB midwi*) OR (TI radiographer* OR AB radiographer*) OR (TI fellow OR AB fellow) OR (TI resident OR AB resident) OR (TI intern OR AB intern) | 1,001.932 |
| 4 | (TI survey* OR AB survey*) OR (TI evaluat* OR AB evaluat*) OR (TI interview* OR AB interview*) OR (TI questionnaire* OR AB questionnaire*) OR (TI assess* OR AB assess*) OR (TI impact OR AB impact) OR (TI validat* OR AB validat*) | 2,447,635 |
| 1 AND 2 AND 3 AND 4 |  | 802 |
|  | Filters: in the last 10 years, English | 631 |

### Scopus search

| Search number | Search string | Result numbers |
| --- | --- | --- |
| 1 | INDEXTERMS ( "Artificial Intelligence" ) OR TITLE-ABS ( "Artificial Intelligence" OR ai OR "Deep Learning" OR "Machine Learning" ) | 1,346,063 |
| 2 | INDEXTERMS ( education ) OR TITLE-ABS-KEY ( educat* OR curricul* OR "professional development" OR teach* OR course* OR fellowship* OR seminar* OR "workshop series" ) | 5,363,546 |
| 3 | INDEXTERMS ( "Health Personnel" ) OR TITLE-ABS ( "health-care work*" OR "health* work*" OR "health-care staff" OR "health* staff" OR "health-care personnel" OR "health* personnel" OR "health-care professional*" OR "health* professional*" OR clinician* OR "health-care student*" OR "health* student*" OR practitioner* OR radiolog* OR physiotherapist* OR "physical therapist*" OR pharmac* OR technician* OR physician* OR "clinical staff" OR doctor* OR "medical staff" OR nurse* OR radiotherapist* OR midwi* OR radiographer* OR fellow OR resident OR intern ) | 4,492,974 |
| 4 | TITLE-ABS ( survey* OR evaluat* OR interview* OR questionnaire* OR assess* OR impact OR validat* ) | 22,102,241 |
| 1 AND 2 AND 3 AND 4 |  | 3,968 |
|  | Filters: in the last 10 years, English  20/8/24 Lee: This can be limited to article, review and conference paper. | 3,588 |

### ERIC search

| Search number | Search string | Result numbers |
| --- | --- | --- |
| 1 | "artificial intelligence" OR "machine learning" OR "deep learning AND health personnel | 334 |
|  | Filters: in the last 10 years, English |  |
