# Supplementary material for: Assessing the effectiveness of artificial intelligence education and training for healthcare workers: a systematic review
Source: BMC Med Educ. 2026 Mar 10;26:549. doi: 10.1186/s12909-026-08969-3 (PMC13045066; doi:10.1186/s12909-026-08969-3)
Supplement: Supplementary file 2 — Supplementary Material 2. [file 12909_2026_8969_MOESM2_ESM.docx]

# Additional file 2 - Data extraction template

| Study details | Author date |
| --- | --- |
|  | Title |
|  | DOI |
|  | Funding |
| Methods | Aim |
|  | Type of study design |
|  | Study methodology |
|  | Country |
|  | Data Analysis |
| Intervention details | Occupation of healthcare workers |
|  | Setting where training or education occurred |
|  | Type of AI training or education |
|  | Was it a formal or informal training program? |
|  | How training or education was delivered? |
|  | Who provided education or training and who instructed? |
|  | Duration, length, and frequency of training |
|  | What were the AI categories or topics taught in the program? |
| Primary outcome | Number of health worker participants being evaluated |
|  | How is AI training and education evaluation reported? |
|  | Evaluation model if used? |
|  | AI literacy prior to education or training (Level 2a, 2b, 3 in Kirkpatrick Barr) |
|  | AI literacy following education of training (Level 2a, 2b, 3 in Kirkpatrick Barr) |
| Secondary outcomes | Reactions and satisfaction to AI training from participants |
|  | Knowing and understanding AI; modification of attitudes and acquisition of knowledge |
|  | Using and applying AI; change in behavior |
|  | Evaluating and creating AI |
|  | Impacts of AI education and training on the health consumer and patient outcomes |
|  | Impacts of AI training and education on health organization practice or care delivery |
|  | Barriers to AI education and training for healthcare workers |
|  | Enablers for AI education and training for health workers |
