# Supplementary material for: Assessing the effectiveness of artificial intelligence education and training for healthcare workers: a systematic review
Source: BMC Med Educ. 2026 Mar 10;26:549. doi: 10.1186/s12909-026-08969-3 (PMC13045066; doi:10.1186/s12909-026-08969-3)
Supplement: Supplementary file 3 — Supplementary Material 3. [file 12909_2026_8969_MOESM3_ESM.docx]

## **Additional file 3 – Mixed Method Appraisal Tool**

| **Study design** | **Study** | **Screening questions** | | **Criteria for the mixed methods appraisal tool** | | | | | **MMAT Score** |
| --- | --- | --- | --- | --- | --- | --- | --- | --- | --- |
| Mixed methods |  | Are there clear research questions? | Do the collected data allow to address the research questions? | Is there an adequate rationale for using a mixed methods study design to address the research question? | Are the different components of the study effectively integrated to answer the research question? | Are the outputs of the integration of qualitative and quantitative components adequately interpreted? | Are the divergences and inconsistencies between quantitative and qualitative results adequately addressed? | Do the different components of the study adhere to the quality criteria of each tradition of the methods involved? |  |
|  | Abid 2024 | Yes | Yes | No | No | Yes | No | No | 1/5 |
|  | BinJala 2024 | Yes | Yes | Yes | Yes | No | Yes | No | 3/5 |
|  | Chadha 2024 | Yes | Yes | No | No | No | Yes | Yes | 2/5 |
|  | Franco D’Souza 2024 | Yes | Yes | Yes | No | No | No | No | 1/5 |
|  | Hedderich 2021 | Yes | Yes | Yes | No | No | Can’t tell | No | 1/5 |
|  | Krive 2023 | Yes | Yes | Yes | Yes | Yes | Yes | Yes | 5/5 |
|  | Reading Turchioe 2024 | Yes | No | Yes | No | No | Can’t tell | No |  |
|  | Richardson 2022 | Yes | No | Yes | Yes | Yes | Can’t tell | No |  |
|  | Tspora 2023 | Yes | Yes | No | Yes | Yes | No | No | 2/5 |
|  | Van Kooten 2024 | Yes | Yes | Yes | Yes | Yes | No | Yes | 4/5 |
| Qualitative |  |  |  | Is the qualitative approach appropriate to answer the research question? | Are the qualitative data collection methods adequate to address the research question? | Are the findings adequately derived from the data? | Is the interpretation of results sufficiently substantiated by data? | Is there coherence between qualitative data sources, collection, analysis and interpretation? |  |
|  | Barbour 2019 | Yes | Can’t tell | Can’t tell | No | No | No | Can’t tell |  |
|  | Bumbach 2024 | Yes | Yes | Yes | No | No | Yes | Yes | 3/5 |
|  | Teferi 2024 | Yes | Yes | Yes | Yes | No | No | Yes | 3/5 |
|  | Van de Venter 2023 | Yes | Yes | Yes | Yes | Yes | Yes | Yes | 5/5 |
|  | Wiggins 2020 | Yes | Yes | Yes | No | Can’t tell | Can’t tell | Can’t tell | 1/5 |
| Quantitative descriptive |  |  |  | Is the sampling strategy relevant to address the research question? | Is the sample representative of the target population? | Are the measurements appropriate? | Is the risk of nonresponse bias low? | Is the statistical analysis appropriate to answer the research question? |  |
|  | Kansal 2022 | Y | Y | Y | Y | Y | Y | Y | 5/5 |
| Quantitative non-randomized |  |  |  | Are the participants representative of the target population? | Are measurements appropriate regarding both the outcome and intervention (or exposure)? | Are there complete outcome data? | Are the confounders accounted for in the design and analysis? | During the study period, is the intervention administered (or exposure occurred) as intended? |  |
|  | Culp 2024 | Yes | Yes | Yes | Yes | Can’t tell | Yes | Yes | 4/5 |
|  | Finkelstein 2024 | Yes | Yes | Yes | Yes | Yes | No | Yes | 4/5 |
|  | Griewing 2024 | Yes | Yes | Can’t tell | Yes | No | Yes | Yes | 3/5 |
|  | Heredia-Negron 2024 | Yes | Yes | Yes | Yes | Yes | Yes | Yes | 5/5 |
|  | Hu 2023 | Yes | Yes | No | Yes | Yes | No | Yes | 3/5 |
|  | Laupichler 2022 | Yes | Yes | No | Yes | No | Can’t tell | Yes | 2/5 |
|  | Lindqwister 2021 | Yes | Yes | No | Yes | Yes | Can’t tell | Yes | 3/5 |
|  | Mishra 2023 | Yes | Yes | Yes | Yes | Yes | Can’t tell | Yes | 4/5 |
|  | Pauwels 2021 | Yes | Yes | Yes | Yes | Can’t tell | No | Yes | 3/5 |
|  | Perchik 2023 | Yes | Yes | Yes | Yes | No | No | Yes | 3/5 |
|  | Taskiran 2023 | Yes | Yes | No | Yes | Yes | No | Yes | 3/5 |
