# Supplementary material for: Assessing the effectiveness of artificial intelligence education and training for healthcare workers: a systematic review
Source: BMC Med Educ. 2026 Mar 10;26:549. doi: 10.1186/s12909-026-08969-3 (PMC13045066; doi:10.1186/s12909-026-08969-3)
Supplement: Supplementary file 4 — Supplementary Material 4. [file 12909_2026_8969_MOESM4_ESM.docx]

# Additional file 4 - Table of trainers, learners and course characteristics

| **Intervention characteristic** | **Categories (number of studies)** | **References** |
| --- | --- | --- |
| Type of training | In-Person Course (n=11)  Online course (n=9)  In-Person Workshop (n=6)  Online Webinar (n=1) | (41, 42, 54, 56-61, 63, 64)  (37, 40, 44, 47-49, 51, 52, 62)  (39, 43, 45, 46, 53, 55)  (50) |
| Occupation of healthcare worker | Doctors (n=12)  Medical Students (n=12)  Radiologist (n=7)  Doctorate Students (n=5)  Researchers (n=5)  Ancillary Healthcare Workers (n=3)  Nurses (n=2)  Nursing Students (n=2)  Administrators (n=1)  Data Scientist (n=1)  Dentist (n=1)  Dental Student (n=1)  Undergraduate Student (n=1) | (39, 40, 42, 45-47, 50, 52, 56, 60, 62, 63)  (37, 39, 40, 47, 48, 50-52, 54-56, 61)  (44, 49, 53, 56, 58, 63, 64)  (41, 43, 47, 55, 57)  (39, 47, 48, 55, 62)  (42, 60, 62)  (46, 60)  (39, 59)  (39)  (48)  (55)  (55)  (55) |
| Level of learner | Entry to practice (n=15)  Post-registration formalized training program (n=9)  Post-registration workplace learning (n=10) | (37, 39, 40, 43, 47, 48, 50-52, 54-57, 59, 61)  (39, 42, 45-47, 50, 53, 55, 56)  (40, 41, 44, 48, 49, 58, 60, 62-64) |
| Training setting | Academia (n=15)  Health System (n=9)  Conference (n=1)  Undefined (n=2) | (37, 40-43, 47, 48, 51, 52, 55, 57-59, 61, 62)  (39, 44, 45, 49, 53, 54, 56, 63, 64)  (46)  (50, 60) |
| Trainer | Physician (n=11)  AI Specialists (n=13)  Data Scientist (n=6)  Educational Leader (n=4)  Researchers (n=6)  Radiologist (n=4)  Administrators (n=3)  Pharmacists (n=2)  Nurse (n=1)  Stakeholders (n=1)  Radiographer (n=1)  Undefined (n=7) | (37, 39, 40, 47, 51-54, 61, 62, 64)  (40, 42, 44, 49, 51, 53, 54, 57, 60-64)  (39, 40, 51, 57, 63, 64)  (44, 51, 52, 57)  (37, 47, 59, 62-64)  (44, 49, 54, 56)  (39, 54, 63)  (43, 51)  (57)  (60)  (62)  (41, 45, 46, 48, 50, 55, 58) |
| Length | Less than 1 Week (n=12)  1-3 Months (n=8)  4-6 Months (n=2)  6-9 Months (n=2)  9-12 Months (n=1)  Undefined (n=2) | (39, 40, 42, 43, 45, 46, 55-58, 61, 63)  (37, 47, 49, 51, 54, 59, 60, 62)  (44, 48)  (52, 53)  (64)  (41, 50) |
| Meeting Frequency | One-Time (n=8)  Daily (n=5)  Weekly (n=7)  Fortnightly (n=1)  Monthly (n=1)  Undefined (n=4) | (39, 40, 42, 46, 50, 55, 57, 63)  (43, 45, 56, 59, 62)  (37, 47, 48, 51, 52, 58, 61)  (44)  (53)  (41, 54, 60, 64) |
| Student assessments | Pre/post survey (n=9)  Group Project (n=5)  Knowledge MCQ (n=3)  Post survey (n=3)  AI Algorithm Development (n=3)  Capstone Project (n=2)  Literature Review (n=1)  Project Proposal (n=1)  Data set analysis (n=1)  Personal Reflection (n=1)  Essay (n=1)  Oral Presentation (n=1)  Undefined (n=4) | (39, 45, 47-49, 53-56)  (43, 47, 51, 57, 61)  (40, 44, 45)  (42, 44, 52)  (37, 41, 58)  (60, 64)  (37)  (37)  (37)  (51)  (62)  (62)  (46, 50, 59, 63) |
